# The Use of Music and Music Therapy in Ameliorating Depression Symptoms and Improving Well-Being in Nursing Home Residents With Dementia

**DOI:** 10.3389/fmed.2018.00287

**Published:** 2018-10-09

**Authors:** Kendra D. Ray, Eva Götell

**Affiliations:** ^1^Planning and Research Department, Menorah Center for Nursing and Rehabilitation, MJHS, Brooklyn, NY, United States; ^2^Ersta Sköndal Bräcke University College, Stockholm, Sweden

**Keywords:** dementia, depression, music therapy, music activities, geriatrics, Alzheimer's disease, aging

## Abstract

**Background:** Studies have shown music therapy can improve depression symptoms in dementia and the use of music activities show promise to have positive impacts on wellbeing. However, few studies show the influence of a music intervention led by certified nursing assistants (CNAs) trained by music therapists to address depression symptoms and wellbeing in individuals with dementia.

**Methods:** Credentialed music therapists (1) administered 2-weeks of music therapy, (2) a 3-days training to CNAs, (3) followed by 2-weeks of music activities, singing and music-with-movement, led by CNAs for 62 nursing home residents with moderate dementia, (4) then measured depression symptoms using the Cornell Scale for Depression. We obtained video consent for 26 of the 62 residents who were video recorded receiving CNA-led music-based caregiving activities. Using the Music in Dementia Scale, over 200 h of video data was observed and raters measured changes in well-being, e.g., levels of enjoyment, mood and engagement in the residents, during the CNA facilitated music activities.

**Results:** A repeated measures ANOVA revealed that mean depression scores differed statistically significantly between time points, *p* ≤ 0.001. Residents' baseline depression symptoms significantly declined following 2 weeks of music therapy, *p* ≤ 0.001, increased during a 2-weeks wash-out period, *p* = 0.389, but appeared to stabilize following the 2-weeks music activity, *p* = 1.00. A video analysis and paired sampled *t*-test demonstrated a significant improvement in wellbeing in residents who engaged in music with movement, *p* = 0.003. Wellbeing improved slightly, but not significantly for residents who participated in the singing intervention, *p* = 0.165.

**Conclusion:** Findings suggest that music therapy can significantly decrease depression symptoms in nursing home residents with dementia. Music activities designed by music therapists and facilitated by CNAs may help sustain the reduction of depression symptoms and improve wellbeing in nursing home residents with moderate to severe dementia.

## Introduction

Currently, one of the most common symptoms associated with dementia is depression (1). People with dementia are at risk for developing depression due to memory loss (2), which may lead to loss of independence and social isolation that can negatively impact wellbeing (3). Music therapy has been shown to be an effective non-pharmacological treatment for depression in dementia (4–7). Incorporating individualized music-based activities may assist in reducing depression symptoms often associated with a dementia diagnosis, improving mood, and quality of life for people with dementia (PWD) (8).

Singing and music-with movement have been found as effective treatments for people with dementia. In music therapy, singing can lead to a sense of wellbeing as indicated by positive self-esteem, a sense of accomplishment and feelings of belonging (9). For caregivers, the use of Music Therapeutic Caregiving, which involves singing while providing care, has been shown to evoke positive emotions, reduce aggression, and create a sense of mutuality (10). Like singing, physical activities like music-with-movement have also been found to reduce depression symptoms (11). Involvement in these activities can lower risk for falls and help maintain motor skills that may aide in maintaining wellbeing for PWD (12).

Depression symptoms in dementia is an ongoing issue as elderly are transitioning to nursing homes and experiencing cognitive decline (13). Although research has shown that many music therapists have developed techniques that may be easily duplicated by care staff to address symptoms in dementia (14, 15), and nursing research has demonstrated positive effects of music incorporated during caregiving (10, 16), few studies show how collaborative efforts between music therapy and nursing may help to maintain reduction of depression symptoms. For this study, we measure the effectiveness of a 2-weeks music therapy intervention followed by a music activity facilitated by certified nursing assistants (CNAs) trained to incorporate singing and music-with-movement into their caregiving activities.

## Methods

This study used an exploratory design, which all participants served as his/her own control to measure the effectiveness of music therapy and music activities on depression symptoms and wellbeing. Using the Cornell Scale for Depression and video analysis, this design aimed to measure changes in depression symptoms and was modeled after previous studies by Buettner and Fitzsimmons (17). We compared changes between baseline depression symptoms with the effects of 2 weeks of music therapy. Then we measured any lingering effects of the music therapy intervention. A 3-days intense training was given to CNAs teaching them to add singing and music-with-movement to their caregiving duties. Finally, we measured any changes found following the 2-weeks, CNA led music activities. This study, part of a larger research project, used over 200 h of video data for analysis of a singing and music-with-movement group to determine any changes in well-being during the music activities facilitated by the CNAs. To protect the validity of the study, the analysts, a music therapist and nurse, did not participate in providing any form of the intervention and had no relationship with the participants.

### Setting and participants

Nursing home residents were recruited via referrals by clinical staff including nurses, social workers and recreation therapists, from three nursing homes in New York. Informed consent was obtained from legal representatives and verbal assent received prior to resident participation. Video consent was gathered for a portion of residents. Approval for this study was provided by New York University School of Medicine Institutional Review Board. Residents were recruited based on the following inclusion criteria: long-term nursing home resident, informed consent from legal representative, mid-stage dementia as measured by Functional Assessment Staging Test (FAST), stable comorbidities, ability to hear with or without assistive device, and no current psychiatric disorders other than dementia or depression. Nursing home residents absent of depression symptoms were excluded. A priori power analysis was conducted to calculate the required sample size for adequate statistical power (18). Using a G-power analysis, with two group *t*-test, equal groups size, equal sigma, it was determined for a large effect size, *d* = 0.80, the total sample required of 70 was required. Seventy participants with depression symptoms were recruited, although eight did not complete the study due to being discharged from the nursing home prior to completion of the study.

### Intervention and training

Participants were provided 2 weeks of music therapy for three times a week. Music therapy sessions ranged from 30 min to 1 h and were held in small groups of four to six people in a common room on the unit where residents resided. Therapy sessions were based on individualized assessments that gathered resident demographics, historical musical preferences and need to address depression symptoms. Two credentialed music therapists used singing, music and movement, using guitar, keyboard, simple rhythm and tonal instruments. Following the 2-weeks period, CNAs were offered a 3-days training course for 7 h each day that was designed based on previous methods for caregivers (19, 20) to address depressive symptoms. The training entailed educating the CNAs on simple music-based approaches, such as tempo, using familiar songs, and creating an amiable environment for caregiving. CNAs were instructed on the use of their voice for singing, provided with simple music-with-movement examples, and received education on the use of recorded music on iPods and portable speakers. The interventions, singing and music-with-movement were chosen by music therapists, based on ease to administer and with intent to improve mood in residents with dementia and depression symptoms. Then, CNAs were instructed to incorporate these activities into their daily caregiving routine.

#### Video recording

Since we aimed to determine the effectiveness of the 3-days training for CNAs, capturing video data allowed us to measure this effectiveness by observing potential changes in levels of response, levels of engagement, and levels of enjoyment. There were a total of 26 videos eligible to measure any potential changes in wellbeing during the CNA facilitated music activities. The music for both the singing and music-with-movement groups was selected by two credentialed music therapists who had success with the use of the songs during music therapy sessions. The therapist prepared playlists using music that residents responded positively to during music therapy. Music was loaded onto an iPod shuffle for use with a light-weight portable speaker. Each CNA was provided one of each. For both the singing and music-with-movement groups, the camera was set up in front of the room to focus on interactions between the residents and CNA facilitating the music activity. No other individuals were present during video recording.

The singing and music-with-movement activities were usually held in the morning following breakfast or in the afternoon after lunch in a private lounge or resident's room. CNAs were instructed to offer these activities two times per week, during care and while overseeing residents in the day room. Duration ranged from 10 to 15 min, depending on the tolerance of the resident. Prior to singing, the CNAs offered large-print song sheets and a small rhythm instrument. Participants were encouraged to sing-a-long to recorded music with CNAs and were offered an opportunity to sing a cappella independently a song of their choice (if able). Many of the residents sang in their first language, which may have included English, Russian, Spanish, or Hebrew. For music-with-movement, CNAs offered participants a choice of colorful scarves, ribbons, or rhythm instruments. Once distributed, they started the music, then with vitality demonstrated movements and informed the residents to copy and follow the movements.

### Instrumentation

The Cornell Scale for Depression (CSD) measures the severity of depression symptoms in people with dementia. This 19-item scale has been confirmed to have a high level of concurrent validity (21) and is a reliable tool for nursing home residents with dementia (22).

The Music in Dementia Assessment Scale (MIDAS) was used to measure wellbeing of nursing home residents in the video data. The scale measures levels of interest, response, initiation, involvement, enjoyment, and major reactions. It has a high therapist inter-rater reliability, but low staff inter-rater reliability (23). The MIDAS requires at least a 10-min session for analysis.

### Analysis

IBM Statistical Package for Social Sciences Statistics software (version 24) was used to perform a repeated measures ANOVA to compare mean Cornell Scale for Depression scores between the baseline, immediately following music therapy intervention, 2-weeks after music therapy and immediately following the music activity.

Using the MIDAS, we carefully analyzed a video-recorded activity (singing or music-with-movement) for each resident between his/her CNA. The video observers rated average changes in wellbeing as demonstrated by levels of interest, response, initiation, involvement and enjoyment during the first 5 min of music and during the most clinically significant 5 min of the music activity (23). Two observers rated video data separately, then shared outcomes with 95% inter rater reliability to confirm results of MIDAS scores.

## Results

The average age of participants for this study was 85.54 ± 8.25 and most were female (*n* = 53). While most participants spoke English, many spoke other languages including Spanish (12.9%) and Russian (12.9%). At baseline, more than half (58.1%) were prescribed antidepressants and all were diagnosed with dementia of varying types including Alzheimer's (22.6%) and Parkinson's disease (4.8%), Table 1. Using the FAST, we found that all participants had mid-stage dementia.

**Table 1:** Participant baseline demographics.

**Variable**	**Category**	***n* Percent**
Gender	Female Male	53 (85.5%) 9 (14.5%)
Race	African American Caucasian	2 (3.2%) 60 (96.8%)
Ethnicity	Hispanic Non-Hispanic	7 (11.3%) 55 (88.7%)
Language	English Spanish Russian Hebrew	45 (72.6%) 8 (12.9%) 8 (12.9%) 1 (1.6%)
Dementia diagnosis	Alzheimer's disease Vascular Parkinson's disease Mixed Other Unspecified	14 (22.6%) 9 (14.5%) 3 (4.8%) 13 (21%) 3 (4.8%) 20 (32.3%)
Antidepressant meds	No Yes	26 (41.9%) 36 (58.1%)

*Mean age = 85.54, range = 58.9–100.0*.

**Figure 1:**
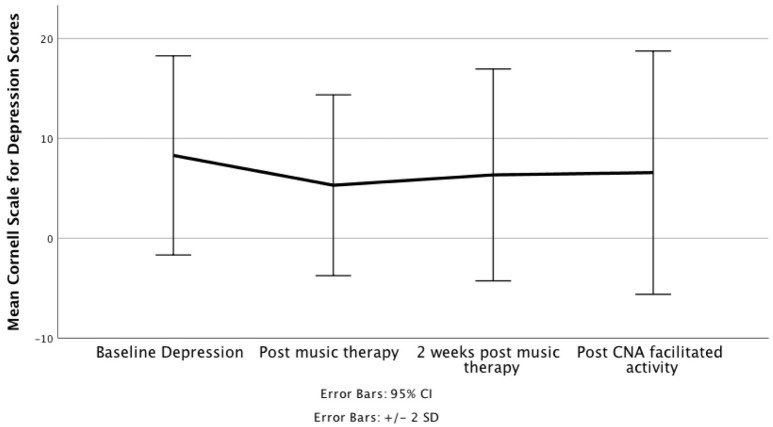
Figure illustrates change over time for depression symptoms in nursing home residents with dementia.

A repeated measures ANOVA revealed that mean Cornell Scale for Depression scores differed statistically significantly between time points. Mauchly's test of sphericity demonstrated that the assumption had been met, *x*^2^(5) = 8.63, *p* = 0.125; therefore, the Greenhouse-Geiser correction showed the results, [*F*_(3, 199.12)_] = 4.90, *p* = 0.004. Residents' depression symptoms significantly declined (8.29 ± 4.99 vs. 5.31 ± 4.53) following 2 weeks of music therapy (*p* ≤ 0.001), increased (5.31 ± 4.53 vs. 6.34 ± 5.30) during a 2-weeks wash-out period (*p* = 0.389) but appeared to stabilize (6.34 ± 5.30 vs. 6.56 ± 6.09) following the 2-weeks music activity (*p* = 1.00). While slight increases in symptoms occurred following music therapy, the increases noted after the CNA facilitated music activities were not significant in comparison with baseline scores (*p* = 0.047), Figure 1.

Of the 62 residents, 26 video consents allowed for video analysis using the MIDAS. Our video analysis and paired sampled *t*-test demonstrated a significant improvement (251.67 ± 132.94 vs. 402.25 ± 113.50) in wellbeing during - the most clinically significant 5 min of the music-with-movement intervention in comparison with the beginning of the intervention, *t*_(11)_ = −3.87, *p* = 0.003. Wellbeing improved slightly, (376.43 ± 167.36 vs. 438.86 ± 55.93) but not significantly for residents who participated in the singing intervention, *t*_(13)_ = −1.47, *p* = 0.165.

## Discussion

The aim of our research was to (1) measure the effectiveness of a music therapy intervention on depression symptoms in nursing home residents with dementia and; (2) measure the effectiveness of a singing and music-with-movement activity facilitated by CNAs on depression symptoms and wellbeing. We found that music therapy significantly decreased depression symptoms and while symptoms began to increase 2 weeks following music therapy, stabilized when CNAs added singing and music-with-movement to their caregiving activities. The 2-weeks washout period appeared sufficient between measurements as we noticed 2 weeks following music therapy, the residents' depression scores begin to increase.

Systematic reviews demonstrate that music therapy and behavioral management techniques, such as caregiver implemented interventions incorporated with appropriate training are the only nonpharmacological interventions shown to significantly reduce behavioral disturbances (like depressive symptoms) in people with dementia (24–26), but since music therapists are not available to nursing home residents 24/7, the Center for Medicare and Medicaid have suggested that diversional activities, such as singing or movement be considered as a nonpharmacological treatment to help manage symptoms of dementia (12). In the current study, while it appears that both the singing and movement activity were helpful in sustaining reductions in depression symptoms, the video analysis demonstrated that the music-with-movement protocol significantly improved well-being in the participants. Our results are similar to Sarkamo (27) and colleagues' research that found improvements in mood for people with dementia whose caregivers sang to them.

### Limitations

The MIDAS, a measurement tool created to rate wellbeing through responses to music, allowed us to capture levels of interest, response and communication that was not accounted for using the Cornell Scale for Depression. While this tool was helpful, the authors acknowledge that it is still being tested for validity. In addition, we were limited in our ability to analyze video for all 62 participants, which reduced our video data sample. Though the sample was reduced, the video provided hundreds of hours of data and visual representations of how the music may have been positively improving wellbeing and sustaining reductions in depression symptoms.

Due to the MIDAS requirements of at least 10 min of interactions for data use, we only analyzed one singing activity and one music-with-movement activity. Shorter videos may have had different outcomes than the results we reported. Future studies should aim to analyze more than one session to compare outcomes over a period of time. An additional approach may have been to compare wellbeing in music therapy vs. music activities using video data. This study lacked a formal control group; therefore, outcomes presented should be interpreted carefully.

## Conclusion

Our study highlights the benefits of providing music therapy and music-based care for nursing home residents on a continuum. The study interventions show an effective example of how music therapy skills can be shared to extend music's therapeutic benefits and sustain reductions of depression symptoms. This research may be translated into clinical settings, which begins treatment with a music therapist who achieves primary goals then transfers instruction and facilitation to a caregiver. We suggest that 2 weeks of music therapy is necessary to assess the impact of the music therapy and to gather information necessary to share with CNAs. This study showed that educated CNAs may choose singing or music-with-movement and expect positive outcomes related to wellbeing, though our preliminary outcomes suggest the latter may be more effective. As the prevalence of depression in dementia increases and nursing home placements continue to rise, methods for treatment as demonstrated in this article are needed to meet the needs of our elderly; though future research is needed to determine the generalizability of our findings. By promoting and leading ongoing training programs in institutional settings, together with nursing professionals, music therapists can provide a quality continuum of care for aging adults with depression in dementia.

## Author contributions

KR wrote the manuscript with support from EG. All authors provided critical feedback that helped to conclude the analysis and findings for manuscript.

### Conflict of interest statement

The authors declare that the research was conducted in the absence of any commercial or financial relationships that could be construed as a potential conflict of interest.
